# Psychotherapeutic interventions in individuals at risk for Alzheimer’s dementia: a systematic review

**DOI:** 10.1186/s13195-021-00956-8

**Published:** 2022-01-31

**Authors:** Ayda Rostamzadeh, Anna Kahlert, Franziska Kalthegener, Frank Jessen

**Affiliations:** 1 Department of Psychiatry and Psychotherapy, University of Cologne, Medical Faculty, 50937 Cologne, Germany; 2 Institute for Psychology, Rheinisch Westfälische Hochschule Aachen, Philosophical Faculty, 52056 Aachen, Germany; 3 German Center for Neurodegenerative Diseases (DZNE), Venusberg Campus 1, Gebäude 99, 53127 Bonn, Germany; 4 Excellence Cluster on Cellular Stress Responses in Aging-Associated Diseases (CECAD), University of Cologne, 50924 Cologne, Germany

**Keywords:** Psychotherapy, Psychoeducation, Prevention, Individuals at risk, Subjective cognitive decline, Mild cognitive impairment, Alzheimer’s disease, Alzheimer’s dementia

## Abstract

**Background:**

Expanding technologies of early detection of Alzheimer’s disease allow to identify individuals at risk of dementia in early and asymptomatic disease stages. Neuropsychiatric symptoms, such as anxiety and depression, are common in the course of AD and may be clinically observed many years before the onset of significant cognitive symptoms. To date, therapeutic interventions for AD focus on pharmacological and life style modification-based strategies. However, despite good evidence for psychotherapy in late-life depression, evidence for such therapeutic approaches to improve cognitive and emotional well-being and thereby reduce psychological risk factors in the course of AD are sparse.

**Methods:**

A systematic review was conducted in PUBMED, PsycINFO, Web of Science, and Clinical Trials to summarize the state of evidence on psychotherapeutic and psychoeducational interventions for individuals at risk for Alzheimer’s dementia. Eligible articles needed to apply a manualized and standardized psychotherapeutic or psychoeducational content administered by trained professionals for individuals with subjective cognitive decline or mild cognitive impairment and measure mental health, quality of life or well-being.

**Results:**

The literature search yielded 32 studies that were included in this narrative summary. The data illustrates heterogeneous therapeutic approaches with mostly small sample sizes and short follow-up monitoring. Strength of evidence from randomized-controlled studies for interventions that may improve mood and well-being is scarce. Qualitative data suggests positive impact on cognitive restructuring, and disease acceptance, including positive effects on quality of life. Specific therapeutic determinants of efficacy have not been identified to date.

**Conclusions:**

This review underlines the need of specific psychotherapeutic and psychoeducational approaches for individuals at risk of Alzheimer’s dementia, particularly in terms of an early intervention aiming at improving mental health and well-being. One challenge is the modification of psychotherapeutic techniques according to the different stages of cognitive decline in the course of AD, which is needed to be sensitive to the individual needs.

**Supplementary Information:**

The online version contains supplementary material available at 10.1186/s13195-021-00956-8.

## Background

Neurodegenerative diseases such as Alzheimer’s disease (AD) have become a major public health challenge. It is estimated that due to the rapidly aging population, the dementia prevalence will rise up to 135.5 million patients in 2050 [1]. Expanding technologies of early disease detection allow biomarker-based diagnosis in the preclinical and prodromal stages, long before functional disability of dementia becomes apparent [2–4]. The preclinical phase of AD comprises the condition of subjective cognitive decline (SCD), where healthy adults are concerned about a cognitive decline, while performance on neuropsychological testing is within normal limits and activities of daily living (ADL) are preserved [3]. Individuals with SCD with a biomarker-based evidence of AD are at higher risk for developing cognitive decline [5–7]. The prodromal phase of AD is the condition of mild cognitive impairment (MCI), which is an at risk state for Alzheimer’s dementia and is defined as a clinical condition, where subjects have mild cognitive decline, but preserved ADL [2], thus not fulfilling dementia criteria. Individuals diagnosed with MCI are a heterogeneous group, with only about 30 % of them developing Alzheimer’s dementia within 3 years after clinical MCI diagnosis [8]. However, MCI patients with a biomarker-based evidence of AD have a high risk of approximately 70% to develop Alzheimer’s dementia within 3 years [9]. Currently, research on early disease identification, dementia risk-prediction, and prevention strategies in pre-dementia stages of AD is carried out with the aim of impacting on modifiable risk factors and targeting molecular pathways of AD to ultimately slow the disease course [10–13]. As epidemiological studies suggest, about one third of dementia cases worldwide can be attributed to potentially modifiable risk factors [14]. Against this background, non-pharmacological prevention strategies are investigated more intensely. Several prevention studies with a multidimensional approach (including physical, lifestyle, cognitive and nutritional interventions) aim to reduce modifying risk factors for AD targeting the primary outcome to improve the cognitive outcome, but essentially leaving behind psychological risk factors for AD [13, 15–21]. Psychological risk factors include neuropsychiatric symptoms (NPS), including anxiety, depression, and sleep disturbance. NPS may accelerate the course of neurodegenerative diseases and are potential modifying risk factors for cognitive decline [21–25]. There seems to be a bi-directional relationship between (sub-) syndromal NPS and cognitive decline. While NPS may enhance cognitive decline and may also be the early manifestation of a pre-dementia-stage of a neurodegenerative disorder, such as AD, cognitive decline in itself may stimulate NPS, particularly due to the psychological burden associated with worsening of cognition. Several studies highlight the profound stress, anxiety, and worries that individuals and close-others encounter shortly after early AD detection [26–28]. For individuals at risk of developing Alzheimer’s dementia, the diagnosis of MCI may increase their uncertainty, as it is associated with an unclear prognosis on the level of an individual. We know from literature that individuals with MCI encounter difficulties in social, psychological, and daily living context, which may lead to depression or anxiety and they specifically ask for information about the causes of the syndrome, the potential disease course, accompanying symptoms, social consequences, and available treatments [26]. However, little is known on how individuals in at risk stages for Alzheimer’s dementia cope with their diagnosis and their impending impairments in the long-term. In the face of an, to a certain degree, unpredictable and still incurable disease like AD, disease acceptance and its consequences are of paramount importance for the patient and their close-others. There is empirical evidence that coping strategies and illness perceptions have a major impact on well-being and quality of life of individuals with chronic diseases [29]. The field of psychooncology has been integrated to the management of cancer patients since the early 1970s. Psychooncology contributes to the clinical care of patients, to the training of personnel in psychological management of cancer patients, to cancer prevention strategies and to the management of psychiatric and psychosocial problems during the continuum of the cancer illness. There is empirical evidence that psychosocial care in oncology helps to alleviate emotional burden and improves well-being in patients and close-others. The psychooncological care follows a stepped approach with a special focus on the individual patients’ needs during the disease course, from the disease prevention, to diagnosis, to therapy and follow-up care. This model could provide the framework for a holistic disease management for patients and their close- others in the continuum of AD, from the early preclinical stage, such as SCD, to the dementia stage, with adapted contents. At the current stage, a comprehensive psychotherapeutic concept with the scope of prevention, self-management, and coping, as well as improving well-being, mental health, and quality of life within the course of Alzheimer’s disease is still lacking.

Non-pharmacological interventions that focus on cognitive function such as the impact of cognitive function on daily living have been widely studied in individuals with MCI. The majority is investigating effects of cognitive training interventions such as cognitive remediation or compensation approaches and moreover physical exercise interventions [30, 31]. There is some evidence that cognitive training and physical interventions may improve cognitive abilities in individuals with MCI; however, the effects on daily functioning are small. There is some ongoing research on non-pharmacological interventions for individuals with SCD, which strengthen the impact of cognitive and psychological interventions to improve mental health such as cognitive and emotional well-being [32, 33].

In summary, data on psychotherapeutic interventions and their effects on mental health and quality of life in early disease stages of AD is sparse. Therefore, the aim of this systematic review is to provide an overview on current concepts for psychotherapeutic and psychoeducational interventions for individuals in early disease stages of AD, such as individuals with SCD and MCI, and their effects on behavioral or psychological outcomes, such as depression, anxiety, or quality of life.

## Methods

### Search strategy

Search strings consisted of three sections that were combined using the Boolean Operator “AND.” One section was referring to the psychotherapy and psychoeducational intervention, the second section was referring to the at risk stages of Alzheimer’s disease “mild cognitive impairment” and “subjective cognitive impairment,” and the third section was referring to Alzheimer’s disease (see Additional file 1 for the detailed search strings). The final search from inception to June 2021 (last read out 09.06.2021) was carried out in PUBMED, PsycINFO, Web of Science, and Clinical Trials. Furthermore, the reference lists of all publications included in this review were hand searched for additional studies. Search strategy, screening, and data selection were carried out in accordance with the PRISMA criteria [34]. This review is registered in the international prospective register of systematic reviews (PROSPERO) with the registration number: CRD42020145399.

### Paper selection/inclusion criteria

We included studies that investigated individuals at risk of developing Alzheimer’s dementia, such as individuals with SCD or MCI. The diagnosis of MCI needed to be defined according to the NIA-AA criteria for mild cognitive impairment or according to the MCI criteria of Petersen 2004 [2, 35]. Since the stage of late MCI and early dementia is often a transition stage, studies that investigated this particular patient group were also included, when they were considered relevant for our research question. Therefore, a number of included studies refer to the transitional stage of late MCI and mild dementia [36–39]. Due to the recent standardization of SCD [40], we decided to broaden the definition of SCD to conceptually equivalent diagnosis, to include as many studies as possible in this review. We used the Jessen et al. [40] criteria to decide, whether the study populations met the criteria for SCD, when authors did not specify the underlying SCD concept. Inclusion criteria were that articles were published in a peer-reviewed journal in English or German language. No restriction regarding the publication date was applied. This review considered all types of study designs including quantitative (such as observational, prospective and retrospective cohort studies, clinical trials, randomized-controlled trial (RCT)), qualitative, and mixed methods designs. To be included, studies had to apply a manualized and standardized psychotherapeutic or psychoeducational content administered by specifically trained professionals and had to measure mood or quality of life as a primary or secondary outcome. The interventions needed to be clearly described.

### Screening and assessment of studies

In the screening process, eligibility based on title and abstract was checked according to the inclusion criteria. These procedures were performed by two independent reviewers. Discrepancies in rating were resolved through discussion, and when necessary, a third reviewer judged the respective publication. In case of an unclear eligibility, a full text review was performed.

### Data extraction

Due to the heterogeneity of study results with regard to intervention type, study length, measuring methods, and outcome measures, we decided to perform a systematic narrative review. In order to ensure a systematic data extraction for the narrative review, an evaluation matrix for data analyses was designed based on the inclusion criteria and our research question. Two independent reviewers performed data analyses and, in case of any discrepancies, a third reviewer re-evaluated. The next steps included extraction of additional information on study design, characteristics, and population and on the main outcome measures. The narrative synthesis included the target population characteristics, the therapeutic interventions, the methodology, the study setting, and the type of outcome. Thematic categories were predefined based on the research question and were further refined during the data analysis process.

### Quality assessment (risk of bias)

The quality of included studies was evaluated by two independent reviewers using the risk of bias tool proposed by Hawker et al. in 2002 (see Table 1) [41]. The tool comprises 9 items (summed score from 10 = very poor to 40 = good) relating to abstract and title, introduction and aims, method and data, sampling, data analysis, ethics and bias, presentation of results, transferability, and usefulness in order to judge the methodological rigor of the studies. Discrepancies between raters were resolved by discussion and where necessary re-assessed by a third reviewer.

**Table 1:** Study characteristics

Study and country	Sample size (***N***)	Follow-up	Characteristics	Intervention	Control	Study design	Outcomes	Main findings	Quality
Psychotherapy for individuals with MCI									
Gildengers et al. 2016 USA	94	3/6/9/12 m post-int.	Patients: *N* = 74 Dg.: MCI Gender: 47 f, 27 m Age: 75 yrs. (M) MMSE: / Caregivers: *N* = 20 Gender: 16 f, 4 m Age: 66.6 yrs. (M)	Problem-solving therapy (PST) with and without moderate-intensity physical exercise (PE)	Usual care enhanced by the same assessments as the intervention group	Single-blinded randomized controlled trial. Couples therapy led by master’s level therapists	− Depression (Prime-MD/Mini) − Anxiety (GAD-7)	Preliminary results: high acceptance for intervention and usefulness in managing stress and cognitive problems	Good
Joosten-Weyn Banningh et al. 2008 Netherlands	46	2w post-int.	Patients: *N* = 23 Dg: MCI Gender: 13f, 10 m Age: 68.7 yrs. (M) MMSE 26.7 (M) Caregivers: *N* = 23 Gender: 12f, 11 m Age: 70.4 yrs. (M)	Combination of cognitive behavioral therapy and psychoeducation	N/A	Non-randomized trial Group therapy led by psychotherapists	− Depression (GDS) − Well-being (SF-36) − Subscales Acceptance and Helplessness (ICQ) − Marital satisfaction (MMQ) − Burden of Caregiver	Preliminary results: high motivation for intervention. Evidence for significant increase of acceptance and a trend for an increased marital satisfaction. The significant others reported an increased awareness of memory and behavioral problems	Good
Joosten-Weyn Banningh et al. 2011, 2013 Netherlands	94	6–8 m post-int.	Patients: *N* = 47 Dg.: MCI Gender: 20 f, 27 m Age: 69.9 yrs. (M) MMSE: 25.7 (M) Caregivers: *N* = 47 Gender: 31f, 16 m Age: 68.5 yrs. (M)	Combination of cognitive behavioral therapy and psychoeducation	Waiting-list	Non-randomized trial Group therapy led by psychotherapists	− Depression (GDS) − Well-being (SF-36) − Subscales Acceptance and Helplessness (ICQ) − Marital satisfaction (MMQ) − Burden of Caregiver	Increase of acceptance in MCI patients was maintained at follow-up, with increased insight into cognitive decline. Increase in sense of competence increased in the significant others. Worse helplessness and well-being at follow-up compared to post-intervention in patients and significant others	Good
Miller et al. 2007 USA	1	N/A	Dg.: MCI Gender: 1 m Age: 80 yrs. MMSE: /	Interpersonal psychotherapy (IPT) for depressed elders	N/A	Individual therapy led by psychiatrists.	− Depression	Standard IPT techniques need to be modified, including active integration of the caregiver into the treatment process	Fair
Scheurich et al. 2008 Germany	24	12 m post-int.	Patients: *N* = 12, Dg.: MCI Gender: 7f, 5 m Age: 66.8 yrs. (M) MMST: 24 (M) Caregivers *N* = 12, Gender: 7f, 5 m Age: 61.5 yrs. (M)	Combination of cognitive behavioral therapy and psychoeducation	N/A	Non-randomized pilot trial Group therapy, no information about the professional background of therapist	− Depression (GDS, BDI) − Life quality (SF-36)	Reduced anxiety, anergia, and withdrawal in MCI patients. Caregivers showed reduced sleep disturbances, irritability, and aggressiveness toward the diseased family member	Good
Tonga et al. 2016 Norway	3	N/A	Patients: *N* = 3 Dg.: mild AD Gender: 2f, 1 m Age: 59 yrs., 66 yrs., 77 yrs. MMSE: 27, 23, 20	Cognitive Rehabilitation and Cognitive behavioral therapy (Cordial Manual) [72]	N/A	Individual therapy led by a psychologist	− Depression (HADS) − Anxiety (HADS) − Client Satisfaction (CSQ-8) − Burden of Caregiver (RSS)	Apathy and anosognosia hindered treatment adherence, while caregivers were essential for treatment and homework completion. Psychotherapy for individuals with AD needs to allow flexibility of the manual, according to the resources and preferences of the patients	Fair
Tonga et al. 2021 Norway	198	4/10 m post-baseline	Intervention group: *N* = 100 Dg.: MCI (*n* = 32) and dementia (*n* = 68) Gender: 45f, 55 m Age: 69.4 (M) MMSE: 24.7 (M) Caregivers: *N* = 100 Gender: 66f, 34 m Age: 66.8 yrs. (M) Control group: *N* = 98 Dg.: MCI (*n* = 48), dementia (*n* = 48) Gender: 47f, 51 m Age: 70.7 yrs. (M) MMSE: 24.5 (M) Caregivers: *N* = 98 Gender: 67f, 31 m Age: 65.7 yrs. (M)	Cognitive Rehabilitation and Cognitive-behavioral therapy (Cordial Manual) [72]	Treatment as usual	Randomized controlled trial Group therapy led by nurses, psychiatrists, occupational therapists and psychologists	− Depression (MADRS) − Neuropsychiatric Inventory − Quality of life (QoL-AD)	Significant improvement in depression within the intervention group compared to the control group. No group differences with regard to neuropsychiatric symptoms or quality of life	Good
Psychoeducational intervention for Individuals with MCI									
Barton et al. 2017 UK	16	8w post-int.	Patients: *N* = 16 Dg.: MCI Gender: 9f, 7 m Age: 74.2 yrs. (M) MMSE: /	Psychosocial group intervention based on the recovery model and psychoeducation	N/A	Non-randomized trial Group therapy led by facilitators trained in group therapy	− Mental Well-Being (Warwick Edinburgh Scale) − Goal Attainment Scale	Well-being improved significantly and satisfaction with the intervention was high	Fair
Bier et al. 2015 (study protocol) Belleville et al. 2018 Canada	145	3/6 m post-int.	Psychosocial intervention group: *N* = 43, Dg.: MCI Gender: 24f, 19 m Age: 72.1 yrs. (M) MMSE: / Cognitive intervention group: *N* = 40 Dg.: MCI Gender: 20f, 20 m Age: 71.3 yrs. (M) MMSE: / Control group: *N* = 44, Dg.: MCI Gender:26f, 18 m Age: 73.1 yrs. (M) MMSE: /	Cognitive intervention according to the MEMO program (MEMO-program) [59] Psychosocial intervention with a CBT approach and psychoeducation	No contact group (no intervention)	Single-blinded randomized controlled trial Group therapy led by therapists (qualified clinicians)	− Depression (GDS) − Anxiety (GAI) − General well-being (GWBS)	No significant effect on mood or well-being in neither group	Good
Diamond et al. 2015 Australia	64	2w post-int.	Intervention group: *N* = 36, Dg.: MCI and/or MDD Gender: 27f, 9 m Age: 67.3 yrs. (M), MMSE: / Control group: *N* = 28, Dg.: MCI and/or MDD Gender: 16f, 12 m Age: 65.6 yrs. (M) MMSE: 28.5 (M)	Multifaceted Healthy Brain Ageing Cognitive Training (HBA-CT) with psychoeducation and computerized cognitive training	Treatment as usual	Single-blinded randomized controlled trial Group intervention led by multidisciplinary specialists (psychiatrists, neurologists, neuropsychologists, clinical psychologists)	− Depression (GDS) − Subjective memory (EMQ) − Pittsburgh Sleep Quality Index (PSQI)	Improvements in self-reported memory, mood, and sleep in the intervention group	Good
Kurz et al. 2009 Germany	40	N/A	Intervention group: Dg.: MCI *N* = 18 Gender: 11f, 7 m Age: 70.4 yrs. (M) MMSE: 27,8 (M) Dg.: mild AD *N* = 10 Age: 66 yrs. 8 M) Gender: 5f, 5 m MMSE: 23.9 (M) Control group: Dg.: MCI *N* = 12 Dg.: MCI Gender: 6f, 6 m Age: 70.8 yrs. (M) MMSE: 28.0 (M)	Cognitive rehabilitation program	Waiting list	Non-randomized trial Group therapy, no information about the professional background of therapist	− Depression (BDI) − Cognition (MMSE) − Activities of daily living (ADL)	Significant improvements on mood and ADL in individuals with MCI	Good
Larouche et al. 2019 Chouinard et al. 2019 Canada	48	3 m post-int.	Intervention group: *N* = 23; Dg.: MCI Gender: 9f, 14 m Age: 71.4 yrs. (M) MMSE: / Control group *N* = 22; Dg: aMCI Gender: 10f, 12 m Age: 70.5 yrs. (M) MMSE: /	Mindfulness-based intervention (MBI)	Psychoeducation-based intervention (PBI)	Single-blinded randomized controlled pilot trial Group intervention led by trained psychologists	− Depression (GDS) − Anxiety (GAI) − Life quality (WHOQOL-Brief and WHOQOL-Brief OLD)	Both interventions had positive effects on anxiety, depression, and age-related QoL	Good
Lu et al. 2013 USA	20	3 m post-int.	Patients: *N* = 10 Dg: MCI Gender: 3f, 7 m Age: 69.2 yrs. (M) MMSE: 27.1 (M) Caregivers: *N* = 10 Gender: 7f, 3 m Age: 66 yrs. (M)	Daily Enhancement of Meaningful Activity (DEMA) intervention with components of problem-solving therapy (PST)	N/A	Non-randomized pilot trial Individual and Couples therapy led by trained nurses	− Depression (PHQ-9) − Well-being (SF-36) − Quality of life (QoL-AD) − Caregiver Burden Scale (CBS)	Evidence for acceptance and feasibility for the program. No significant effects on depression, quality of life and caregiver burden	Good
Lu et al. 2016 Ellis et al. 2019 USA	72	3 m post-int.	Intervention group Patients: *N* = 17 Dg.: MCI Gender: / Age: 71.6 yrs. (M) MMSE: / Caregivers: *N* = 17 Gender: / Age: 65.5 yrs. (M) Control group: Patients: *N* = 19 Dg: MCI Gender: / Age: 76.8 yrs. (M) MMSE: / Caregivers: *N* = 19 Gender: / Age: 70.8 yrs. (M)	Daily Enhancement of Meaningful Activity (DEMA) intervention with components of problem-solving therapy (PST)	Information support attention control group	Randomized controlled pilot trial Individual and couples therapy led by trained nurses	− Depression (PHQ-9)	No significant effect on mood in neither group. The intervention group indicated significantly higher usefulness, ease of use, and total satisfaction than the control group. No significant group difference in the caregivers’ ratings regarding satisfaction with the treatment	Good
Rovner et al. 2012, 2016, 2018 USA	221	6/12/18/24 m post-int.	Intervention group: *N* = 111 Dg: MCI Gender: 86 f, 25 m Age: 75.5 yrs. (M) MMSE: 25.8 (M) Control group: *N* = 110 Dg: MCI Gender: 89 f, 21 m Age: 76.2 yrs. (M) MMSE: 25.6 (M)	Behavioral activation therapy: a manual-based behavioral treatment to increase cognitive, physical and/or social activity	Supportive therapy offered a structured, nondirective psychological treatment	Single-blinded randomized controlled trial Individual intervention led by trained community health workers	− Depression (GDS) − Quality of life	No significant group difference on depression in both treatment groups	Good
Schmitter-Edgecombe et al. 2014 USA	46	3 m post-int.	Intervention group: *N* = 23 care-dyads Patients: Dg.: MCI Gender: 16f, 7 m Age: 72.96 yrs. (M) MMSE: / Control group: *N* = 23 care-dyads Patients: Dg: MCI Gender: 11f, 12 m Age: 73.35 yrs. (M) MMSE: /	Cognitive rehabilitation multi-family group intervention, including problem-solving therapy and psychoeducation	Standard care	Randomized controlled trial Group intervention led by trained clinical psychology doctoral students and community professionals (i.e., psychologists, social workers)	− Quality of Life-Alzheimer’s disease (QOL-AD) − Depression (GDS) − Coping (CSE)	No significant group differences on psychological measures. Caregivers reported improved coping behavior	Good
Smith et al. 2017 (study protocol) Chandler et al. 2019 USA	272	6/12/18 m post-int.	Patients: *N* = 272 Dg.: MCI Gender: 112f, 160 m Age: 75 yrs. (M), MMSE: 28.36 (M)	Mayo Clinic Healthy Action to Benefit Independence and Thinking (HABIT) program, a 50-h group intervention including psychoeducation, memory compensation training, computerized cognitive training, yoga, patient and partner support groups, and wellness education	N/A	Multisite, cluster randomized trial Group intervention led by therapist (neuropsychologists, dementia educators, exercise specialists, nurse practitioners, social workers)	− Quality of Life-Alzheimer’s disease (QOL-AD) − Depression (CES-D) − Modified chronic disease Self-Efficacy Scale	No significant effects on the outcomes could be determined in neither intervention group by 12 months. Wellness education had a greater effect on mood than computerized cognitive training, and yoga had a greater effect on activities of daily living than support groups at 12 months. Cognitive training had the least effect on these outcomes	Good
Wells et al. 2013, 2019 USA	14	2 m post-baseline	Intervention group: *N* = 9 Dg: MCI Gender: / Age: 73 yrs. (M) MMSE: 27 (M) Control group: *N* = 5, Dg: MCI Gender: / Age: 75 yrs. (M), MMSE: 27 (M)	Mindfulness Based Stress Reduction (MBSR), standardized mindfulness meditation intervention, with psychoeducation on stress and stress relief	Waiting list	Randomized controlled pilot trial Group intervention, no information about the professional background of therapist	− Quality of Life-Alzheimer’s disease (QOL-AD) − Depression (CES-D) − Perceived Stress Scale (PSS) − Resilience Scale (RS) − Mindful Attention Awareness Scale (MAAS)	No significant group differences with regard to psychological outcomes. The qualitative interviews revealed positive perceptions of class attendance, development of mindfulness skills, including meta-cognition, importance of the group experience, enhanced well-being, shift in MCI perspective, decreased stress reactivity and increased relaxation, improvement in interpersonal skills	Fair
Psychotherapy for Individuals with SCD									
N/A									
Psychoeducational intervention for Individuals with SCD									
Cohen-Mansfield et al. 2015 Israel	44	10 weeks post-baseline	Health promotion: *N* = 15, Dg.: SCD Gender: 13f, 2 m Age: 74.44 yrs. (M), MMSE: 28.67 (M) Cognitive Training: *N* = 15, Dg.: SCD Gender: 9f, 6 m Age: 72.8 yrs. (M), MMSE: 27.93 (M) Participation-centered: *N* = 14, Dg.: SCD Gender: 10f, 5 m Age: 73.21 yrs. (M) MMSE: 28.93 (M)	Health promotion course: psychoeducation on health behaviors and lifestyle modification; dementia and delirium; age-related cognitive decline and MCI, such as cognitive activities to keep the mind fit. Cognitive training course: the Advanced Cognitive Training for Independent and Vital Elderly (ACTIVE) with focus on memory, reasoning, and speed of processing Participation centered course: CBT-based delivery of memory, cognitive, and organizational strategies	Waiting list	Single-blinded randomized controlled pilot trial Group intervention, no information about the professional background of therapist	− Well-being (UCLA Loneliness Scale) − Depression (GDS)	All three interventions resulted in significant improvement in cognitive function as measured by the computerized cognitive assessment. Self-report of memory difficulties decreased significantly in the cognitive training group participants. All approaches seemed to decrease loneliness	Good
Hoogenhout et al. 2012 Netherlands	50	4w post-int.	Intervention group: *N* = 24 Dg.: SCD Gender: 24f Age: 66.0 yrs. (M) MMSE: 29.24 (M) Control group: *N* = 26 Dg.: SCD Gender: 26f Age: 66.1 yrs. (M) MMSE: 29.11 (M)	Psychoeducation about cognitive aging and contextual factors (negative age stereotypes, beliefs, health and lifestyle), focusing on skills and compensatory behavior	Waiting list	Randomized controlled trial Group intervention, no information about the professional background of therapist	− Maastricht Metacognition Inventory (MMI)ESQ − Psychological Well-being Quotient (PWQ)	Participants in the experimental group reported less emotional reactions towards cognitive functioning than participants in the control condition. The intervention improved an important aspect of metacognition. No significant differences between the groups in psychological well-being	Good
Marchant et al. 2018 (study protocol) Marchant et al. 2021 Multi-center (France, Germany, Spain, UK)	147	2/6 m post-baseline.	Intervention group: *N* = 73 Dg.: SCD Gender: 47f, 26 m Age: 72.1 yrs. (M) MMSE: 28.7 (M) Control group: *N* = 74 Dg.: SCD Gender: 48f, 26 m Age: 73.3 yrs. (M) MMSE: 28.9 (M)	Mindfulness based approach for seniors [76] with psychoeducational components	Health self-management program to promote engagement in activities to improve health and well-being	Multi-center, observer-blind randomized controlled trial Group interventions led by clinically trained facilitators (mindfulness-based teachers, clinical psychologist or equivalent degree)	− Anxiety (STAI) − Depression (GDS) − Emotion regulation − Mindfulness (FFMQ) − Life quality (WHOQOL-Brief) − Well-being (Loneliness Scale) − Pittsburgh Sleep Quality Index (PSQI)	No significant group differences with regard to psychological outcomes. Both interventions showed a reduction in trait anxiety on follow-up	Good
Smart et al. 2016 Smart and Segalowith 2017 Canada	38	2w post-int.	Patients: *N* = 15 Dg.: SCD Gender: 11f, 4 m Age: 69.6 yrs. (M) MMSE: 28 (M) Control: *N* = 23 Dg.: healthy control Gender: 9f, 14 m MMSE: 27.78 (M)	Mindfulness Based Stress Reduction (MBSR) based on Kabat-Zinn, standardized mindfulness meditation intervention, with psychoeducation on stress and stress relief	Psychoeducation on cognitive aging	Single-blinded randomized controlled pilot trial Group intervention, no information about the professional background of therapist	− Depression (GDS) − Mindfulness (FFMQ) − Anxiety (AMAS) − Negative mood regulation (NMR)	No significant group differences with regard to psychological outcomes. Both interventions improved psychological findings (reduction of cognitive complaint, reduction of anxiety and self-judgment of one’s own mental functioning)	Good

*AD* Alzheimer’s disease; *ADL* Activities of Daily Living; *AMAS* Adult Manifest Anxiety Scale; *BDI* Beck Depression Inventory; *CBS* Caregiving Burden Scale; *CES-D* Center of Epidemiology Depression Scale; *CSE* Coping Self-efficacy scale; *CSQ-8* Client Satisfaction Scale; *Dg.* diagnosis; *f*, female; *FFMQ* Five-Facet Mindfulness Questionnaire; *FU* follow-up; *GAD-7* Generalized Anxiety Questionnaire; *GAI* Geriatric Anxiety Inventory; *GDS* Geriatric Depression Scale; *GSE* General Self-Efficacy Scale; *GWBS* General Well-Being Schedule; *HADS* Hospital Anxiety and Depression Scale; *ICQ* Illness Cognition Questionnaire; *m* male; *M* mean; *MAAS* Mindful Attention Awareness Scale; *MADRS* Montgomery–Asberg Depression Rating Scale; *MBSR*, mindfulness based stress reduction; *MCI* mild cognitive impairment; *MDD* major depressive disorder; Maudsley Marital Questionnaire; *MMSE* Mini Mental State Exam; *MoCA* Montreal Cognitive Assessment; *MSEQ* Memory Self-Efficacy Questionnaire; *N*, number; *NMR* Negative Mood Regulation Scale; *PHQ* Patient Health Questionnaire; *post-int.* post-intervention; *PSQI* Pittsburgh Sleep Quality Index; *PSS* Perceived Stress Scale; *QoL* Quality of Life; *QOL-AD*, Quality of Life in Alzheimer’s disease; *RSS* Relatives’ Stress Scale; *SCD* subjective cognitive decline; *SF-36* Short Form Health 36; *STAI* State-Trait Anxiety Inventory; *yrs* years; *w* week; *WHOQOL* World Health Organization Quality of Life Brief scale

## Results

### Included studies

**Fig. 1:**
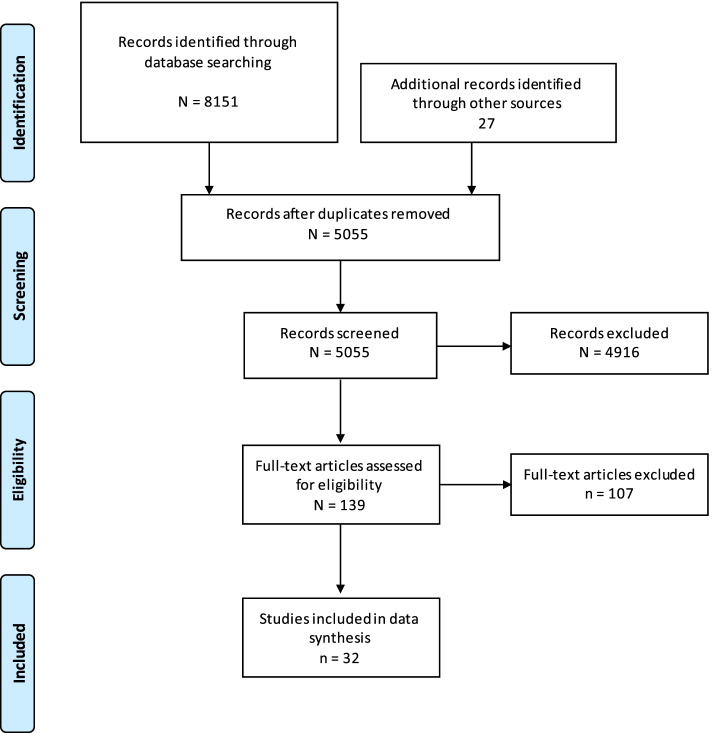
PRISMA Flow-Chart of database search

The initial search yielded 8151 papers. Twenty-six additional articles were identified through reference check. One hundred thirty-seven articles were selected for full text review. After full text review, 32 publications fulfilled the inclusion criteria for analysis. The detailed selection process according to the PRISMA criteria is depicted in Fig. 1 [34].

The 32 included papers are summarized in Table 1. Furthermore, some of the included publications referred to the same study and are marked as so in Table 1. Of the included papers, 13 originated from the USA [42–54], followed by 6 from Canada [55–60], 4 from the Netherlands [61–64], 2 from Germany [37, 38], 2 from Norway [36, 39], and one from each of the following countries: Australia [65], Israel [66], and UK [67]. Two publications referred to a multi-center study [68, 69], which was performed in France, Germany, Spain, and the UK. Among the 6 papers focusing on interventions for individuals with SCD, none explored the effects of a manualized psychotherapeutic intervention, but all offered psychoeducational interventions in addition to mindfulness-based stress reduction or health promotion and cognitive training courses. All were carried out within randomized controlled trials [55, 56, 61, 66, 68, 69]. A total of 26 papers referred to interventions with individuals with MCI. Amongst them, 8 papers described manualized psychotherapeutic interventions [36, 37, 39, 42, 43, 62–64] and 18 papers described psychoeducational interventions in addition to cognitive rehabilitation, cognitive training, mindfulness-based stress reduction, behavioral activation, or a recovery model approach [38, 44–54, 57–60, 65, 67]. The majority of studies included short-term (up to 12 weeks post intervention) or immediate post-intervention follow-up assessments. Long-term follow-up assessments (6 or more months post-intervention) were described in 14 publications [37, 39, 43, 48, 49, 51–53, 59, 60, 62, 63, 68, 69].

### Systematic narrative review

#### Psychotherapy in individuals with MCI

A total of five research groups have described psychotherapeutic interventions in individuals with mild cognitive impairment [36, 37, 39, 42, 43, 62–64].

Three research groups have chosen a cognitive behavioral therapy (CBT)-based approach and investigated the therapeutic effects in follow-up assessments, ranging from immediate post-intervention up to 12 months follow-up [36, 37, 39, 62–64]. With regard to therapeutic effects on mood in MCI patients, mixed findings were reported. Smaller non-randomized studies showed no significant effects, neither in the short-term (*n* = 94) [63] nor in the long-term follow-up (*n* = 24 [37], *n* = 94 [63]), whereas one recently published paper with a larger sample (*n* = 198) of a randomized-controlled study showed a significant reduction of depressive symptoms in the intervention group (cognitive rehabilitation and cognitive behavioral therapy) as compared to the treatment as usual control group by 6 months post-intervention (*p* < 0.001) [39]. The cognitive rehabilitation and cognitive behavioral treatment program comprised CBT, reminiscence therapy, and cognitive rehabilitation. This study, however, did not find any significant group changes with regard to overall neuropsychiatric symptoms or quality of life.

With regard to feelings of helplessness and well-being, Banningh et al. reported significantly worse findings on both scales 6–8 months post-intervention in all participants as compared to immediate post-interventional assessments (*p* < 0.05) [63]. Furthermore, disease acceptance in patients was maintained improved at 6-8 months follow-up (*p* < 0.001).

Preliminary and confirmatory findings from other studies reveal, that CBT-, problem-solving-therapy (PST)-, and interpersonal therapy (IPT)-based interventions are well accepted by and satisfying for participants, if the psychotherapeutic techniques are modified for the needs of the addressed population [37, 42, 43].

#### Psychoeducational interventions in individuals with MCI

A total of 10 research groups described psychoeducational interventions in addition to cognitive rehabilitation [38, 50], cognitive training [52, 53, 59, 60, 65], mindfulness-based stress reduction (MBSR) [46, 47, 57, 58], behavioral activation [44, 45, 48, 49, 51, 54], or enriched by a recovery model approach [67], with follow-up assessments ranging from immediate post-intervention to 24 months follow-up.

With regard to mood, well-being and quality of life no therapeutic effects in MCI patients, neither at short-term (3 months) nor at long-term (24 months) follow-up, were detected in most studies, including four randomized controlled trials [48–51, 54, 59, 60] and one randomized non-controlled trial [52, 53]. The studies had mostly large samples and described psychoeducational interventions enriched with different approaches, ranging from cognitive rehabilitation (*n* = 46 [50] to behavioral activation (*n* = 72 [54], *n* = 221 [48, 49, 51]).

Cognitive training was applied by two research groups in addition to psychoeducation: the Canadian researchers used the MEMO program [70], which included episodic memory strategies as well as exercises to increase attentional control (*n* = 145 [59, 60]) and the US researchers followed the “Healthy Actions to Benefit Independence and Thinking” program with computer-based cognitive training (*n* = 272 [52, 53]).

Immediately post-interventional assessments in smaller randomized controlled (*n* = 64 [65]) and non-randomized waiting-list controlled (*n* = 40 [38]) studies, described significant improvements in depressive symptoms (*p* < 0.01 [38]; *p* = 0.01 [65]), subjective memory functioning (*p* = 0.03 [65]), and sleep quality (*p* = 0.01 [65]) in the intervention group as compared to a control group. Significant improvements in well-being 2 months after intervention as compared to baseline (*p* < 0.01) were reported by Barton et al. (*n* = 16) [67].

Qualitative data from one research group indicated a high acceptability and feasibility of a multi-component Daily Enhancement of Meaningful Activity (DEMA) intervention, including psychoeducation, planning of meaningful activities, dealing with negative emotions and coping strategies, with improvement in meaningful activities and satisfaction in the intervention group as compared to the control group at 3 months follow-up [44, 45].

Two research groups addressed the effects of psychoeducation-based interventions with MBSR-based therapy on MCI patients within randomized controlled trials (*n* = 48 (Chouinard et al. 2019; Larouche, Hudon, and Goulet 2019), *n* = 14 [46, 47]). The Canadian research group concluded that at 3 months follow-up, equivalent beneficial effects on depression (*p* = 0.03), anxiety (*p* = 0.02), and age-related quality of life (*p* = 0.02) were detected in both the intervention and control group [57, 58]. Furthermore, improved problem-focused coping strategies, particularly in active coping, were detected in both groups. The results were confirmed by research of Wells et al. [46, 47], where additional qualitative interviews with participants of the MBSR group revealed the development of mindfulness skills, benefits of the group experience, enhanced well-being, shift in MCI perspective, decreased stress reactivity, and increased relaxation and improvement in interpersonal skills.

#### Psychotherapy in individuals with SCD

Manualized psychotherapeutic interventions for individuals with SCD were not detected in the included studies.

#### Psychoeducational interventions in individuals with SCD

A total of four research groups described psychoeducational interventions alone [61] or in combination with cognitive training and CBT-based interventions [66] and MBSR-based interventions [55, 56, 68, 69] with follow-up ranging from immediately post-intervention to 6 months follow-up.

With regard to depression and well-being, significant therapeutic effects on individuals with SCD were neither reported by Cohen-Mansfield et al. (*n* = 44), describing three different intervention types (psychoeducational health promotion, cognitive training and a CBT-based participation-centered course), nor by Hoogenhout et al. (*n* = 50), following an exclusively psychoeducational approach. However, at 10 weeks follow-up, a trend on decreasing loneliness was detected in all three intervention groups, and self-reported memory difficulties were reduced significantly (*p* ≤ 0.05) in the study of Cohen-Mansfield et al. [66]. Hoogenhout et al. confirmed significant fewer negative emotional reactions toward cognitive functioning immediately post-intervention in the intervention group as compared to controls (*p* = 0.004) [61].

Two research groups investigated the effects of psychoeducation with MBSR-based therapy on individuals with SCD (*n* = 147 [68, 69], *n* = 38 [55, 56]). Marchant et al. conducted a multi-center randomized-controlled trial to investigate the impact of a MBSR intervention on psychological outcomes in comparison to a health self-management program in individuals with SCD. The authors concluded that no group differences were detected with regard to psychological outcomes at follow-up. However, both interventions showed a reduction in subclinical trait anxiety immediately post-intervention and at 6 months follow-up. The results were similar to a smaller study by Smart et al. [55, 56], where immediately post-intervention in both groups a trend in decrease in cognitive complaints, increase in memory self-efficacy, reduction in self-reported anxiety, and self-judgment of one’s own mental functioning was detected.

## Conclusions

This systematic narrative review showed that studies on the effects of psychotherapeutic approaches for individuals at risk of Alzheimer’s dementia are limited. While reviews about this topic have been published before [32, 33, 71], we think this systematic review contributes insight to the current state of literature, as it (i) includes only trials that used standardized and manualized psychotherapeutic or psychoeducational interventions and (ii) covers the full spectrum of individuals at risk for Alzheimer’s dementia, including individuals with SCD and MCI.

This review comprises more studies on therapeutic interventions for MCI patients than for individuals with SCD. While a RCT with a large sample (*n* = 198) of MCI patients showed a significant reduction of depressive symptoms in the intervention group (cognitive rehabilitation and cognitive behavioral therapy) as compared to the treatment as usual control group 6 months post-intervention [39], non-randomized CBT-based trials with smaller sample sizes but longer follow-up assessments of 6–12 months did not find any effects on depressive symptoms (*n* = 24 [37], *n* = 94 [63]). Although psychoeducational interventional studies with small sample sizes detected some positive immediate post-interventional therapeutic effects on well-being (*n* = 16 [67]) and mood (*n* = 64 [65]; *n* = 40 [38]), this review underlines that the majority of existing evidence from randomized controlled (*n* = 145 (Belleville et al. 2018; Bier et al. 2015); *n* = 72 (Ellis, Altenburger, and Lu 2019); *n* = 221 (Rovner et al. 2012, 2018; Rovner and Casten 2016); *n* = 46 (Schmitter-Edgecombe and Dyck 2014)) and randomized non-controlled (*n* = 272 (Chandler et al. 2019; Smith et al. 2017)) trials with mostly large cohorts and longitudinal follow-ups, ranging from 3 to 24 months, does not confirm these findings.

With regard to psychotherapeutic interventions, no data in individuals with SCD were identified, while data regarding psychoeducational approaches addressing individuals with SCD are available from four research groups [55, 56, 61, 66, 68, 69]. The only study that was performed in a randomized controlled manner and in a large cohort of individuals with SCD revealed effects of both an MBSR-based intervention and a health self-management program, on mental health and quality of life at 6 months follow-up, but no group differences [69]. The study, however, showed a significant reduction of trait anxiety post-intervention in both groups, intervention and control, that was maintained at 6 months follow-up.

Literature indicates that individuals with cognitive impairment, such as MCI, need highly individualized psychotherapeutic interventions, as these impairments interfere with the ability to adopt new coping skills, problem-solving skills, and transfer acquired skills to everyday life [36, 72]. This review depicts that psychotherapeutic and psychoeducational interventions for older adults in pre-dementia stages are feasible and may suggest that the degree of cognitive impairment in the pre-dementia stages may not necessarily influence the ability to learn skills such as psychotherapeutic or mindfulness interventions [39, 42, 46, 47, 62, 63]. Informatively, the qualitative ratings of perceived benefit and understanding of the intervention were not correlated with baseline cognition, which suggests that the degree of cognitive impairment in MCI may not influence the ability to learn skills in a therapeutic intervention [46, 47].

As an example how to tailor the therapy manuals to individuals with cognitive impairments, Tonga et al. described the experiences with and the required adjustments of a Cognitive Rehabilitation and Cognitive-behavioral treatment manual for early dementia in Alzheimer’s disease (CORDIAL) [72] within a case-control study with MCI and mild dementia patients [36]. The cognitive behavioral treatment with elements of cognitive rehabilitation and reminiscence methods were completed by homework assessments to promote transfer of novel behaviors into the everyday context. The authors stressed that it is crucial to be flexible with the manual regarding the individual needs of the patients and their caregivers and to consider the caregivers’ impact on completion of the homework and the adherence to the treatment. They concluded that therapists need to take into account possible disease-related barriers such as anosognosia or apathy, which might hinder treatment adherence; therefore, the patient’s motivation and disease awareness are even more important for ensuring treatment adherence, than the presence of a caregiver. Indeed, the patients’ insight into their cognitive impairments is a necessary requirement for a successful psychotherapy. Banningh et al. described that significant improvements of the insight into illness by MCI patients might be achieved by tailored cognitive behavioral therapies [63].

Early interventions in preclinical and prodromal AD within the scope of a treatment to improve mental health, disease-acceptance, and life quality might have a secondary effect in terms of slowing cognitive decline and therefore reducing the risk or delaying the onset of dementia. Several preventive non-pharmacological strategies have been conducted, some still ongoing, but there is still limited evidence to support a cause-effect relationship between a single preventive strategy such as physical exercise, stress reduction, nutrition, and treatment of psychiatric co-morbidities and the development or progression of dementia. There are several studies that followed a multifactorial intervention approach, including regular exercise and healthy diet, reduction of vascular risk factors, psychosocial stress, and major depressive episodes, amongst them the “Finnish Geriatric Intervention Study to Prevent Cognitive Impairment and Disability (FINGER),” the “Multidomain Alzheimer Preventive Trial (MAPT),” the “Prevention of Dementia by Intensive Vascular Care (preDIVA),” the “SCD-Well” trial as part of the “Medit-Ageing” project (Silver Santé Study), and the “Body, brain, life for cognitive decline (BBL-CD)” [13, 15–20, 68]. These interventions may be the most promising strategy for the prevention of cognitive decline and the development of individualized therapeutic interventions for the different stages of cognitive decline. However, only to a significantly lesser degree psychological risk factors and non-cognitive outcomes, such as mental health and quality of life, are primarily addressed in most of the prevention studies, leaving this field largely unexplored.

Mindfulness-based therapy, for instance, can help to promote acceptance and reduce maladaptive cognitive emotion regulation strategies, such as ruminating. Especially acceptance-related non-judgment and non-reaction to irritative factors seem to alleviate psychological distress and may be an approach in interventions for individuals with SCD and even for MCI patients. Literature indicates that cognitive restructuring may reduce subjective memory complaints, whereas memory training may improve objective memory function [32, 33]. One way to promote these skills are mindfulness-based interventions, which have been developed from the mindfulness-based stress reduction program by Dr. Jon Kabat-Zinn [73]. There is data that MBSR is feasible in individuals with MCI and that the level of cognitive decline and memory impairment do not necessarily mean an inability to learn mindfulness intervention skills [46, 47]. Furthermore, MBSR has stress-reducing effects, improves well-being, and might improve acceptance and awareness of cognitive decline, which is of major concern for those facing cognitive decline and fear of developing dementia. In this context, the technique of expectancy modification [33] might be an interventional approach for treatment of individuals with SCD or MCI. The expectancy towards one’s own cognitive performance and cognitive competence can be improved by cognitive restructuring, e.g., during psychotherapeutic sessions, and psychoeducation by changing beliefs and attitudes about experienced memory impairment [33, 74]. Though existing quantitative data did not show significant effects on mood of MBSR-based interventions as compared to control conditions [46, 47, 55–58, 68, 69], additional qualitative data revealed positive findings on other outcomes, such as mindfulness skills, enhanced well-being, decreased stress reactivity, and increased relaxation [46, 47]. This leads to the phenomenon that findings from qualitative data, such as a high satisfaction and perceived benefit, are not mirrored in the quantitative assessments [75]. One explanation might be that subtle changes in mood or well-being might be missed by measurement with solely quantitative scales.

Overall, only limited conclusions about the efficacy of the cited studies can be drawn due to insufficiently rigorous study designs, short follow-up times, varying sample sizes ranging from 1 to 272, heterogeneous therapeutic techniques, and outcome measures. Findings on effects on mental health and well-being are therefore diverging and comparing the effects of different psychotherapeutic techniques as well as psychoeducational interventions on mood and quality of life is intricate. As some research groups report likewise effects of intervention and control conditions, it remains open, if the effects are attributable to specific types of interventions, treatment moderators, or other factors, such as participating in a study, interacting with a group, or being supported by a facilitator. Of note, in the cited papers, psychiatric comorbidities, such as depression or anxiety disorder, were exclusion criteria, and the majority of participants were not significantly depressed or anxious at baseline and well-being was generally rated medium to high; hence, there was little chance for the interventions to improve mood and well-being, as measured by quantitative scales. To conclude, the current literature reveals that approaches of psychotherapeutic and psychoeducational interventions are addressed in research projects and underline the feasibility of these interventions, but to date, robust data from RCT’s with large sample sizes providing evidence for significant therapeutic effects on mental health, quality of life and well-being are rare.

Given the strong evidence for psychoeducational interventions and psychotherapy in the field of psychiatric disorders and psychooncology, this field should be opened up systematically for neurodegenerative disorders, such as AD. Psychoeducation provides systematic disease-specific information, such as early recognition and management of disease symptoms, and general information, such as promotion of healthy lifestyle, improving self-management, and disease acceptance. Determinants of psychotherapy are, amongst others, resource activation, actualization of the patient’s problems, motivational clarification, and improving problem-solving skills. In the course of demographic changes, more emphasis should be placed on psychological conditions affecting the elderly, particularly on those who suffer from subjective or objective cognitive decline and actively seek professional help, as their perceived impairments may cause psychosocial stress and are often accompanied by the fear of dementia. A more holistic approach of preventive care with a stepped psychological-based AD management program for individuals which face AD diagnosis would therefore empower them to actively cope with their diagnosis and possible prognosis, than to wait for the disease progression. Moreover, these non-pharmacological interventions are associated with less side effects and are more cost-effective than medications. A future course of action in AD would be to arise awareness for the necessity of longitudinal RCT's addressing mental health and metacognitive abilities for individuals in preclinical and prodromal stages of AD that follow a mixed-method approach, with quantitative outcome measures and complementary qualitative evaluations to gain a deeper understanding of the benefits and possible limits of the interventions.

## Supplementary Information

**Additional file 1.** Search strings.

## Data Availability

Not applicable.

## Abbreviations

AD: Alzheimer’s disease
ADL: Activities of daily living
CBT: Cognitive behavioral therapy
CORDIAL: Cognitive Rehabilitation and Cognitive-behavioral treatment for early dementia in Alzheimer disease
DEMA: Daily Enhancement of Meaningful Activity
e.g.: For example
IPT: Interpersonal psychotherapy
MBSR: Mindfulness based stress reduction
MCI: Mild cognitive impairment
MT: Mindfulness training
*n*: Number
NIA-AA: National Institute on Aging – Alzheimer's Association
NPS: Neuropsychiatric symptoms
PRISMA: Preferred reporting items of systematic review and meta-analyses
PST: Problem-solving-therapy
RCT: Randomized-controlled trial
SCD: Subjective cognitive impairment
