# Correction to: Psychotherapeutic interventions in individuals at risk for Alzheimer’s dementia: a systematic review

**DOI:** 10.1186/s13195-022-00987-9

**Published:** 2022-03-22

**Authors:** Ayda Rostamzadeh, Anna Kahlert, Franziska Kalthegener, Frank Jessen

**Affiliations:** 1 Department of Psychiatry and Psychotherapy, University of Cologne, Medical Faculty, 50937 Cologne, Germany; 2 Institute for Psychology, Rheinisch Westfälische Hochschule Aachen, Philosophical Faculty, 52056 Aachen, Germany; 3 German Center for Neurodegenerative Diseases (DZNE), Venusberg Campus 1, Gebäude 99, 53127 Bonn, Germany; 4 Excellence Cluster on Cellular Stress Responses in Aging-Associated Diseases (CECAD), University of Cologne, 50924 Cologne, Germany

**Correction to: Alz Res Ther 14, 18 (2022)**

10.1186/s13195-021-00956-8

Following the publication of the original article [1] the authors noticed that first and family names of all authors were swapped. The original article [1] has been updated.
