# Supplementary material for: Psychotherapeutic interventions in individuals at risk for Alzheimer’s dementia: a systematic review
Source: Alzheimers Res Ther. 2022 Jan 31;14:18. doi: 10.1186/s13195-021-00956-8 (PMC8802419; doi:10.1186/s13195-021-00956-8)
Supplement: Supplementary file 1 — Additional file 1. Search strings. [file 13195_2021_956_MOESM1_ESM.docx]

Additional file 1 Search Strings

| **PubMed**  ((Psychosocial[All Fields] OR ("psychotherapy"[MeSH Terms] OR "psychotherapy"[All Fields]) OR (psychoeducation)) AND ("mild cognitive impairment"[All Fields] OR MCI[All Fields] OR "subjective cognitive decline"[All Fields] OR "subjective cognitive impairment"[All Fields] OR "subjective Memory decline"[All Fields] OR "subjective Memory impairment"[All Fields] OR ("scd"[All Fields]) OR ("sci"[All Fields]) OR ("risk"[MeSH Terms] OR "risk"[All Fields]) OR prodromal[All Fields]) AND (("alzheimer disease"[MeSH Terms] OR ("alzheimer"[All Fields] AND "disease"[All Fields]) OR "alzheimer disease"[All Fields] OR "alzheimer's"[All Fields]) OR "Alzheimer's disease"[All Fields])) |
| --- |
| **Web of Science**  ((Psychosocial) OR (psychotherapy) OR (psychoeducation)) AND ((mild cognitive impairment) OR (MCI) OR (subjective cognitive decline) OR (subjective cognitive impairment)OR(subjective Memory decline) OR (subjective Memory impairment) OR (scd) OR (sci) OR (risk) OR (prodromal)) AND ((Alzheimer disease) OR (Alzheimer's) OR (Alzheimer's disease)) |
| **PsycInfo**  ((Psychosocial) OR (psychotherapy) OR (psychoeducation)) AND ((mild cognitive impairment) OR (MCI) OR (subjective cognitive decline) OR (subjective cognitive impairment)OR(subjective Memory decline) OR (subjective Memory impairment) OR (scd) OR (sci) OR (risk) OR (prodromal)) AND ((Alzheimer disease) OR (Alzheimer's) OR (Alzheimer's disease)) |
| **ClinicalTrials**  ((Psychosocial) OR (psychotherapy) OR (psychoeducation)) AND ((mild cognitive impairment) OR (MCI) OR (subjective cognitive decline) OR (subjective cognitive impairment)OR(subjective Memory decline) OR (subjective Memory impairment) OR (scd) OR (sci) OR (risk) OR (prodromal)) AND ((Alzheimer disease) OR (Alzheimer's) OR (Alzheimer's disease)) |
